# A Remarkable Meeting

**Published:** 1919-04-05

**Authors:** 


### IRISH NURSING AFFAIRS.

### A Remarkable Meeting.

THE Irish nursing world has suddenly found itself confronted with the economic problem. It was presented by Miss Louie Bennett at the meeting which she recently addressed at the Dublin Mansion House, already described. The first-fruits of this meeting was another public meeting, held on Monday of this week at the Royal College of Surgeons' premises, St. Stephen's Green. This meeting was summoned by the Irish Nurses' Association, and was crowded, a large number of persons being obliged to stand. Mr. J. B. Story, President of the Royal College of Surgeons, presided, and Miss Reeves, Matron of Steeven's Hospital, opened the proceedings with an address, proposing a complete revolution in nurses' hours and nurses' pay. The meeting was most interesting. All the hierarchy of the profession were present, including a few of the most reactionary "leaders." This is the first time on record that any established Society of Irish nurses ever met to inform the public that the women who nurse the sick poor of the country have an economic grievance. The "leaders" have been converted suddenly and *en masse*!

The soul of the meeting was Colonel Sir Arthur Chance, R.A.M.C., one of Dublin's most popular figures. Although a prominent member of the Irish Nursing Board, Sir Arthur showed himself to be completely above the party factions which have brought shame on the profession in Ireland. Nay, to these he unhesitatingly attributed the chaos that prevails, and made a powerful appeal for union. Mrs. Mortished, Secretary to the newly-formed Nurses' Trade Union, rose to speak, and Sir Arthur Chance courteously invited her to step up to the platform, "so that her valuable contribution to the proceedings might be heard by all." A short but telling speech was made by Miss McLaughlin, a nurse in training, who referred to the long hours which seem never-ending, and to the utterly worn-out condition which they produce, so that at times to bring even a glass of water to a suffering patient becomes an intolerable burden.

Sir John Lynch, a prominent Dublin citizen, spoke on behalf of Hospital Governors, not officially, but from what he regarded as their point of view. The burden of his remarks was that the nurse's claims had never yet been presented to Hospital Committees. Plenty of criticism, said Sir John, but no specific request, no consonant voice. And then he offered nurses a sound piece of advice. Get together a Committee of ten or a dozen, consisting of nurses, and let it not consist of matrons only. Let this Committee represent no factions, but the whole profession, and let a demand on Hospital Governors be formulated, based on an expert knowledge of the necessities of the sick. Sir Arthur Chance followed, and with the skill of a statesman formally proposed a resolution, which was seconded and carried, to the effect that a public meeting of nurses should be held in the immediate future for the purpose of electing such a Committee. Every nurses' society, including the College and the Union, Sir Arthur stated should be included, the presidents of which should supervise the electing of the Committee.

This was a most remarkable meeting, including prominent members of the medical profession, Hospital Governors, members of the Irish Nurses' Association, the Irish Nurses' Trade Union, and the College of Nursing. The proposition to elect a Common Committee and draw up a scheme of hours and salaries for nurses appeared to receive unanimous approval.

